# Atorvastatin Improves Ventricular Remodeling after Myocardial Infarction by Interfering with Collagen Metabolism

**DOI:** 10.1371/journal.pone.0166845

**Published:** 2016-11-23

**Authors:** Karla Reichert, Helison Rafael Pereira do Carmo, Anali Galluce Torina, Daniela Diógenes de Carvalho, Andrei Carvalho Sposito, Karlos Alexandre de Souza Vilarinho, Lindemberg da Mota Silveira-Filho, Pedro Paulo Martins de Oliveira, Orlando Petrucci

**Affiliations:** 1 Laboratory of Myocardial Ischemia/Reperfusion, Faculty of Medical Science, State University of Campinas - UNICAMP, Campinas, SP, Brazil; 2 Department of Surgery, Discipline of Cardiac Surgery, Faculty of Medical Science, State University of Campinas - UNICAMP, Campinas, SP, Brazil; 3 Department of Internal Medicine, Faculty of Medical Science, State University of Campinas - UNICAMP, Campinas, SP, Brazil; 4 Section of Pediatric Cardiothoracic Surgery, Department of Surgery, Washington University School of Medicine, St. Louis, Missouri, United States of America; Scuola Superiore Sant'Anna, ITALY

## Abstract

**Purpose:**

Therapeutic strategies that modulate ventricular remodeling can be useful after acute myocardial infarction (MI). In particular, statins may exert effects on molecular pathways involved in collagen metabolism. The aim of this study was to determine whether treatment with atorvastatin for 4 weeks would lead to changes in collagen metabolism and ventricular remodeling in a rat model of MI.

**Methods:**

Male Wistar rats were used in this study. MI was induced in rats by ligation of the left anterior descending coronary artery (LAD). Animals were randomized into three groups, according to treatment: sham surgery without LAD ligation (sham group, n = 14), LAD ligation followed by 10mg atorvastatin/kg/day for 4 weeks (atorvastatin group, n = 24), or LAD ligation followed by saline solution for 4 weeks (control group, n = 27). After 4 weeks, hemodynamic characteristics were obtained by a pressure-volume catheter. Hearts were removed, and the left ventricles were subjected to histologic analysis of the extents of fibrosis and collagen deposition, as well as the myocyte cross-sectional area. Expression levels of mediators involved in collagen metabolism and inflammation were also assessed.

**Results:**

End-diastolic volume, fibrotic content, and myocyte cross-sectional area were significantly reduced in the atorvastatin compared to the control group. Atorvastatin modulated expression levels of proteins related to collagen metabolism, including MMP1, TIMP1, COL I, PCPE, and SPARC, in remote infarct regions. Atorvastatin had anti-inflammatory effects, as indicated by lower expression levels of TLR4, IL-1, and NF-kB p50.

**Conclusion:**

Treatment with atorvastatin for 4 weeks was able to attenuate ventricular dysfunction, fibrosis, and left ventricular hypertrophy after MI in rats, perhaps in part through effects on collagen metabolism and inflammation. Atorvastatin may be useful for limiting ventricular remodeling after myocardial ischemic events.

## Introduction

Heart disease is the leading cause of death in developed countries. Ischemic events in cardiac tissue can lead to cellular, molecular, and interstitial changes that modify the architecture and geometry of the ventricles. This process of ventricular remodeling [1, 2] involves changes at both the site and remote areas of the infarct [3]. Cardiac extracellular matrix (ECM) is composed of structural proteins, such as proteoglycans, glycosaminoglycans, fibroblasts, and collagen, and regulatory proteins, such as matricellular proteins that regulate interactions between cells and ECM [4–6]. The predominant component of the ECM, collagen is synthesized and secreted by cardiac fibroblasts [5, 7]. Collagen types I and III (COL I and COL III, respectively) are the most abundant collagen types in cardiac tissue, together accounting for 95% of the total collagen [2, 7, 8]. Ventricular remodeling is unfavorable to the myocardium in part because it is associated with accumulation of collagen and other ECM components [9].

Matrix metalloproteinases (MMPs) are proteolytic enzymes who functions are directed towards the degradation of collagen and ECM components [10–14]. MMPs are synthesized and secreted by cardiomyocytes, fibroblasts, endothelial cells, and cells involved in inflammatory processes, such as macrophages, neutrophils, and lymphocytes [10]. Elevated MMP levels have been observed after ischemic injury in the myocardium [15]. During tissue remodeling, tissue inhibitors of metalloproteinases (TIMPs) are secreted and regulate the activity levels of MMPs [16, 17]. A dynamic balance between the action and inhibition of MMPs is essential to ensuring control of the degradation and synthesis of collagen and, thus, to maintaining ECM integrity [15]. In addition to MMPs, the inflammatory process has an important role in ventricular remodeling by modulating healing and ECM properties [18]. Several mediators are activated after ischemic insult [19], leading to the infiltration of various inflammatory cells, including those residing in the myocardium as well as macrophages, neutrophils, and monocytes, and the secretion of cytokines, such as interleukin 1 beta (IL1β) and tumor necrosis factor alpha (TNFα) [20].

3-Hydroxy-3-methylglutaryl coenzyme A reductase inhibitors, also known as statins, are drugs indicated for the treatment and prevention of dyslipidemia. Statins stabilize atherosclerotic plaques in cardiovascular disease. Their pleiotropic effects include antioxidant and anti-inflammatory activities, improvement of endothelial function, and reduction of cytokine expression [9, 21, 22]. Atorvastatin has shown beneficial effects in the inhibition of cardiac fibroblasts *in vitro*, the reduction of fibrosis, and the expressions of COL I and COL III [21, 23, 24]. Atorvastatin has been observed to reduce the effects and symptoms of heart failure, which is a deleterious consequence of ventricular remodeling [23, 25]. However, the signaling pathways and molecular mechanisms by which atorvastatin influences ventricular remodeling after myocardial infarction (MI) are not well understood. Thus, the purpose of this study was to determine whether administration of atorvastatin for 4 weeks would influence collagen metabolism and ventricular remodeling in an experimental model of MI in rats.

## Methods

The study was approved by the ethics committee on animal use at the University of Campinas (protocol 2860–1). The study was conducted in accordance with the Guide for the Care and Use of Laboratory Animals (NIH Publication No. 85–23, Revised 1996).

### Animals and induction of MI

Four-week-old male Wistar rats, weighing approximately 180 grams, were used for this study. MI was induced in rats by ligation of the left anterior descending coronary artery (LAD). Briefly, anesthesia was induced by inhalation of 2% isoflurane without intubation, followed by thoracotomy and gentle chest compression to expose the heart. A 6–0 polypropylene suture was passed around the LAD, which was then occluded. The heart was returned to the thoracic cavity, and the chest was closed quickly. Animals were observed during recovery and received acetaminophen (single dose 50 mg/kg, by gavage).

Animals were randomized into three groups. Animals in the sham group (n = 14) were subjected to the surgical procedure without LAD ligation. Animals in the control group (n = 27) were subjected to LAD ligation and received 2 ml of saline solution by oral gavage daily for 4 weeks. Animals in the atorvastatin group (n = 24) were subjected to LAD ligation and received 10mg atorvastatin/kg/day (Lipitor, Pfizer) diluted with 2 ml of saline solution by oral gavage daily for 4 weeks. Dose of atorvastatin used in this study can be considered safe, according to studies evaluating its use in rat models [9, 23, 24]. Animals received the first dose of atorvastatin (or saline solution, for the control group) on the same day as the surgical procedure, after recovery. At the end of 4 weeks, animals were sacrificed under deep anesthesia using an overdose of ketamine (75 mg/kg) and xylazine (15mg/kg), followed by exsanguination performed through inserting a needle into the left ventricular (LV) cavity and aspirating the blood. Hemodynamic, histological, and molecular evaluations were performed.

### Hemodynamic assessment

Hemodynamic data of animals were assessed through an invasive procedure at the end of the 4 weeks. Animals were anesthetized with xylazine (5 mg/kg) and ketamine (75 mg/kg) through intraperitoneal injection. At the end of the procedure the animals were euthanized by receiving additional dose of xylazine (15 mg/kg) and ketamine (75 mg/kg). An SPR-838 pressure-volume catheter (Millar Instruments) was inserted into the cavity of the left ventricle (LV) through the left carotid artery. Pressure and volume of the LV were monitored continuously for correct catheter positioning. The catheter was coupled to a PowerLab 8/30 A/D converter (AD Instruments). Parallel conductance correction was determined by injection of 20 μL of 30% hypertonic saline solution. At the end of the hemodynamic measurements, LV volume correction was performed by using heparinized blood from the animal in a cuvette calibration procedure.

Data of the left ventricle end-diastolic volume (EDV), end-systolic volume (ESV), isovolumic relaxation constant (Tau), maximum derivative of pressure (Max dP/dt), minimum derivative of pressure (Min dP/dt), preload recruitable stroke work (PRSW), and end-systolic PV relationship (ESPVR) were recorded.

### Histological evaluations

After hemodynamic data were obtained, animals were killed and dissected to remove the heart. The LV was separated from other cardiac structures, sectioned into three segments, fixed in10% paraformaldehyde, and embedded in paraffin. Histological 4-μm-thick sections were made. Sections were stained with Masson trichrome for analysis of fibrosis and Picrosirius red for analysis of collagen. All measurements were limited to the papillary muscle as the segment of choice for analysis. The proximal portion of the LAD is the origin of the septal branch. The septal branch is responsible for irrigation of the papillary muscles and is not affected by coronary occlusion, thereby guaranteeing maintenance of these muscles [26]. LV and remote region of the infarct were analyzed. To establish fibrosis in the remote area, the contralateral wall of the LV was identified, considering the region opposite to the infarcted area. For quantitative analysis, the infarcted region was excluded, and just the remote region was analyzed. Optical microscope with polarized light (Imager A2 Axio Carl Zeiss) was used to obtain images of histological sections. Microscopy was performed with 2.5x magnifying lenses and a coupled camera in the microscope (Axio Cam ICC 1, Zeiss). Fragments were reconstructed to form a panoramic image by using PTGui 9.1.3 (Rotterdam, Netherlands). Areas of fibrosis and collagen deposition were analyzed by Image ProPlus6.0 (Rockville, MD).

### Myocyte cross-sectional area

As an indicator of the level of ventricular hypertrophy after MI, myocyte cross-sectional area was evaluated as described by Stefanon *et al*.,[27]. Analysis was performed in a remote region of the LV, by using an optical light microscope (Imager A2 Axio Carl Zeiss) with a 40x magnifying lens. Assessment was randomized by a blinded examiner for all groups. Histological 4-μm-thick sections were obtained and stained with hematoxylin and eosin (H&E), followed by the evaluation of 12–15fields. Cross-sectional area was measured manually by counting 70 cells per animal (~5 cells per field). All analyzed cells exhibited structural integrity of the nucleus and cytoplasm. Image ProPlus6.0 was used to evaluate cell size (in μm).

### Immunoblotting

Immunoblotting was used to evaluate protein expression levels in remote regions of the infarcts. Tissue samples were obtained, placed in liquid nitrogen, and kept at -80°C. Total protein was extracted by RIPA buffer containing protease and phosphatase inhibitors [28]. Samples were homogenized and centrifuged. Supernatant was used to determine the amount of total protein with the BCA Protein Assay Kit (23225/23227, Thermo Scientific). Forty micrograms of protein were applied to an SDS-PAGE gel, electrophoresis was performed, and protein bands were transferred by Trans-Blot^®^ (Turbo-BioRad) to a 0.2-μm nitrocellulose membrane (BioRad).

Proteins were blocked with blocking solution for 2 hours and incubated with primary antibodies for MMP-1/8 (sc30069), TIMP1 (ab61224), COL I (sc8784), COL III (sc28888), procollagen C-proteinase enhancer (PCPE, sc730022), secreted protein acidic and rich in cysteine (SPARC,ab61383), tenascin C (TN-C, ab108930), I kappa B alpha (IκBα, sc371), phosphorylated I kappa B alpha (pIκBα, sc8404), Toll-like receptor 2 (TLR2, ab108998), and TLR4 (ab30667). Primary antibodies were obtained from Santa Cruz Biotechnology and Abcam. Horseradish peroxidase-conjugated anti-rabbit or anti-goat secondary antibodies (Thermo Scientific) were added and incubated for 2 hours, followed by a revealing solution (Super Signal Chemiluminescence Solution and West Pico Chemiluminescent Substrate, Pierce). Images of bands corresponding to proteins of interest were obtained through a photo document system (Gel Logic Imaging System) and analyzed by densitometry. All signs of bands corresponding to proteins of interest were normalized to Ponceau staining [28, 29].

### Quantitative real-time PCR

RNA was extracted from a remote region of the LV by using Trizol reagent (Ambion RNA, Life Technologies). Total RNA was quantified from the 260-/280-nm ratio on the Epoch Micro-Volume Spectrophotometer System. For the reverse transcriptase reaction (cDNA), 1μg of total RNA was used with the High Capacity cDNA Reverse Transcription kit (Applied Biosystems), in accordance with the manufacturer's protocol. Gene expression was measured by quantitative real-time PCR with TaqMan commercially available hydrolysis probes (Applied Biosystems) for *IL-1*, *IL-6*, *NF-κB p50*, and *NF-κB p65*. Expression levels of target genes were normalized to the expression level of actin as a reference gene.

### Statistical analysis

Statistical analyses were performed by using GraphPadPrism for Windows version 5. Continuous variables were expressed as the mean ± standard deviation (SD). All samples were tested for normality by the D’Agostino–Pearson test. Comparison among three groups was performed by one-way analysis of variance (ANOVA) followed by the Bonferroni or Kruskal–Wallis test, when appropriate. P<0.05 was considered statistically significant.

## Results

### Atorvastatin attenuates ventricular dysfunction

Atorvastatin treatment for 4 weeks after LAD ligation significantly reduced EDV compared to the control group. The atorvastatin group had lower ESV and higher Min dP/dT, PRSW, and ESPVR values compared to the control group, but these differences were not statistically significant (Table 1).

**Table 1:** Hemodynamic Data for Rats in the Three Groups of the Study.

Parameter	Sham	Control	Atorvastatin	P value
EDV (μL)	176.2 ± 27.51	220.2 ± 31.67	172.8 ± 36.21^#^	0.04
ESV (μL)	165.3 ± 26.09	201.8 ± 27.70	167.1 ± 31.70^#^	0.05
Tau (ms)	10.51 ± 1.74	20.43 ± 6.90*	18.11 ± 3.56*	<0.01
Max dP/dt (mmHg/s)	7976 ± 1268	6926 ± 2828	5405 ± 1651	0.13
Min dP/dt (mmHg/s)	-8462 ± 1902	-4719 ± 1836*	-5059 ± 2122*	<0.01
PRSW	35.34 ± 20.13	28.97 ± 16.68	40.79 ± 36.57	0.71
ESPVR	4.18 ± 1.54	0.55 ± 0.49*	1.38 ± 1.62*	<0.01

Data were recorded at the end of 4 weeks and are expressed as mean ± SD.

* indicates P<0.05 versus sham group;

^#^ P<0.05 versus control group.

### Atorvastatin reduces fibrosis after MI

**Fig 1:**
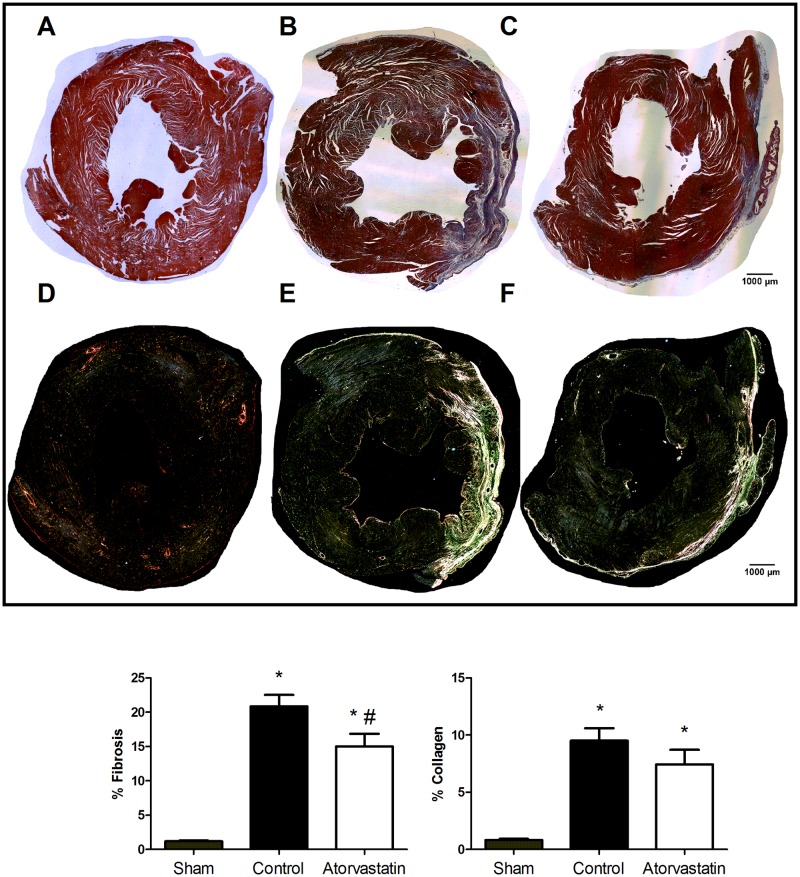
Histological Images Showing LVs of Animals in the Three Groups. Sham (**A** and **D**), control (**B** and **E**), and atorvastatin groups (**C** and **F**). Sections were stained with Masson trichrome for fibrosis analysis (top) or Picro Sirius red for collagen analysis (bottom). Scale bars: 1000μm. Histograms show that the percentage of fibrosis in the LV was reduced after treatment with atorvastatin (white), without affecting the collagen content, compared to the control group (black). Sham group (gray) showed less collagen deposition compared to the atorvastatin and control groups. Data are expressed as the mean ± SD. *P<0.05 versus sham group, #P<0.05 versus control group.

We quantified areas of fibrosis and collagen deposition on the LV in the three groups (Fig 1). Animals subjected to LAD ligation showed larger areas of fibrosis compared to animals subjected to sham surgery (sham: 1.1% ± 0.3%, control: 20.8% ± 7.6%, atorvastatin: 15.0% ± 6.7%; P<0.01). Treatment with atorvastatin for 4 weeks reduced the area of fibrosis compared to the control group (P = 0.03). We found smaller areas of collagen deposition in the LVs of rats in the sham group compared to groups with LAD ligation (sham: 0.81% ± 0.31%, control: 9.5% ± 5.0%, atorvastatin: 7.4% ± 4.5%; P<0.01). No significant difference in the extent of collagen deposition was observed between the control and atorvastatin groups (P = 0.24).

**Fig 2:**
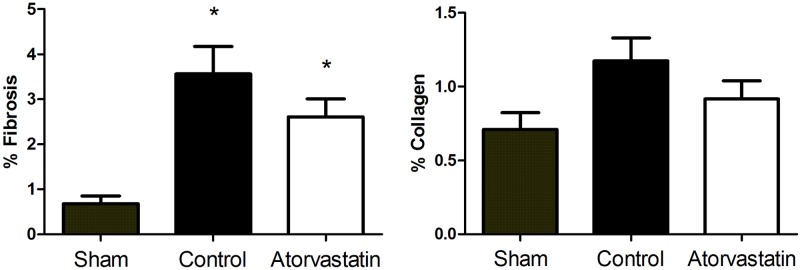
Fibrosis and Collagen Levels in Remote Regions of the LV after MI. Histogram shows that treatment with atorvastatin (white) did not reduce the deposition of fibrosis or collagen compared to the control group (black). Fibrosis content was reduced in animals from the sham group (gray) compared to animals subjected to MI (atorvastatin and control groups). Data are expressed as the mean ± SD. *P<0.05 versus sham group. Data are representative of 14 animals per group.

We evaluated the areas of fibrosis and collagen in remote infarct regions for rats in the three groups (Fig 2). Animals in the sham group had smaller areas of fibrosis compared to animals in the control or atorvastatin group (sham: 0.6% ± 0.4%, control: 3.5% ± 2.7%, atorvastatin: 2.6% ± 1.4%; P<0.01). No significant difference in collagen deposition area was found among the three groups (sham: 0.7% ± 0.3%, control: 1.1% ± 0.7%, atorvastatin: 0.9% ± 0.4%; P = 0.14).

### Atorvastatin reduces ventricular hypertrophy

**Fig 3:**
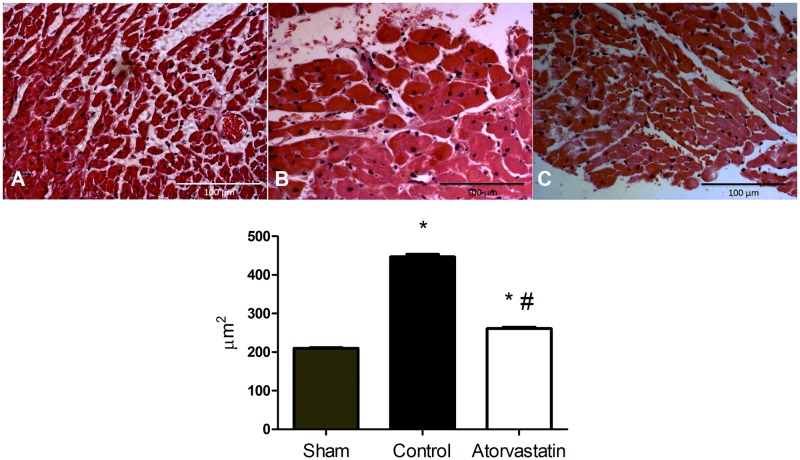
Myocyte Cross-sectional Areas in Remote Regions of LV Infarcts. Images correspond to sham (**A**), control (**B**), and atorvastatin groups (**C**). Scale bars: 100μm. Sections were stained with H&E. Seventy cells were analyzed per plate (n = 14 animals per group). Data are expressed as the mean ± SD. *P<0.05 versus sham group, #P<0.05 versus control group.

To evaluate the extent of ventricular hypertrophy, we analyzed the myocyte cross-sectional area in histological sections of remote regions of the infarcts from animals in the three groups (Fig 3). After 4 weeks, we found significantly smaller myocyte cross-sectional areas in the sham compared to the control or atorvastatin group (sham: 209.6 ± 45.34 μm, control: 446.8 ± 133.0 μm, atorvastatin: 261.1 ± 79.35 μm; P<0.01), as well as in the atorvastatin compared to the control group (P<0.01).

### Atorvastatin modulates collagen metabolism

**Fig 4:**
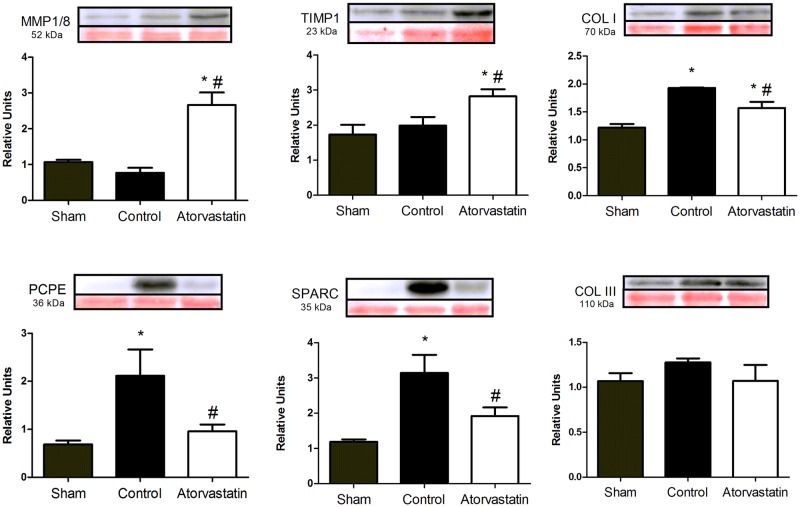
Immunoblotting Results for Expression Levels of Proteins Related to Collagen Metabolism in Remote Regions of LV Infarcts. Representative blots for expression levels of each studied protein. Bands corresponding to each protein (top) were normalized by Ponceau staining (red bands on bottom). Histograms show expression levels determined by densitometry. Data are expressed as the mean ± SD. *P<0.05 versus sham group; #P<0.05 versus control group. Data are representative of nine animals per group.

We studied expression levels of proteins related to collagen metabolism (i.e., MMP-1/8, TIMP1, COL I, COL III, PCPE, and SPARC) in remote regions of LV infarcts from animals in the three groups (Fig 4). The atorvastatin group showed significantly higher MMP-1/8 and TIMP1 but lower COL I expression levels compared to the control group. We found significantly lower expression levels of PCPE and SPARC in the atorvastatin group compared to the control group. The COL III level did not significantly differ among the three groups. Taken together, these results show that atorvastatin treatment strongly influenced collagen metabolism in the LV infarcts.

### Atorvastatin reduces inflammatory response

**Fig 5:**
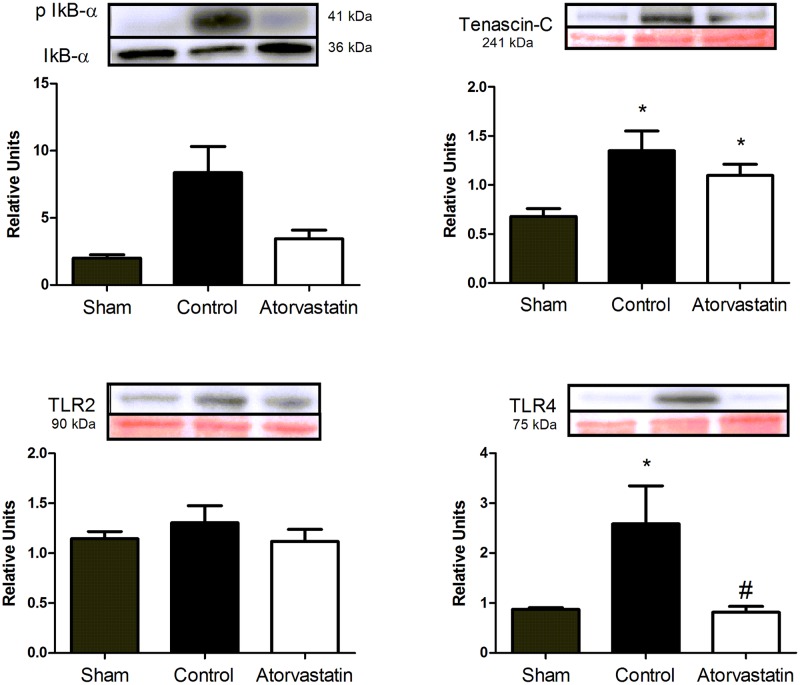
Immunoblotting Results for Expression Levels of Proteins Related to Inflammatory Processes in Remote Regions of LV Infarcts. Representative blots for expression levels of studied proteins. Signs of bands corresponding to phosphorylated IκBα (top) were normalized by total IκBα expression (bottom). Signs of bands corresponding to Tenascin-C (TN-C), TLR2, and TLR4 (top) were normalized by Ponceau staining (red bands on bottom). Histograms show expression levels determined by densitometry. Data are expressed as the mean ± SD. *P<0.05 versus sham group, ^#^P<0.05 versus control group. Data are representative of nine animals per group.

Using immunoblotting, we analyzed expression levels of several potent mediators involved in the inflammatory response after MI (Fig 5). Treatment with atorvastatin influenced the activity (phosphorylation) of IκBα, but this difference was not significant among the groups. TN-C showed lower expression levels in animals from the sham compared to the other two groups, but the difference between the atorvastatin and control groups was not significant. We found lower expression levels of TLR4 in the atorvastatin compared to the control group, but no significant difference between the groups for TLR2.

**Fig 6:**
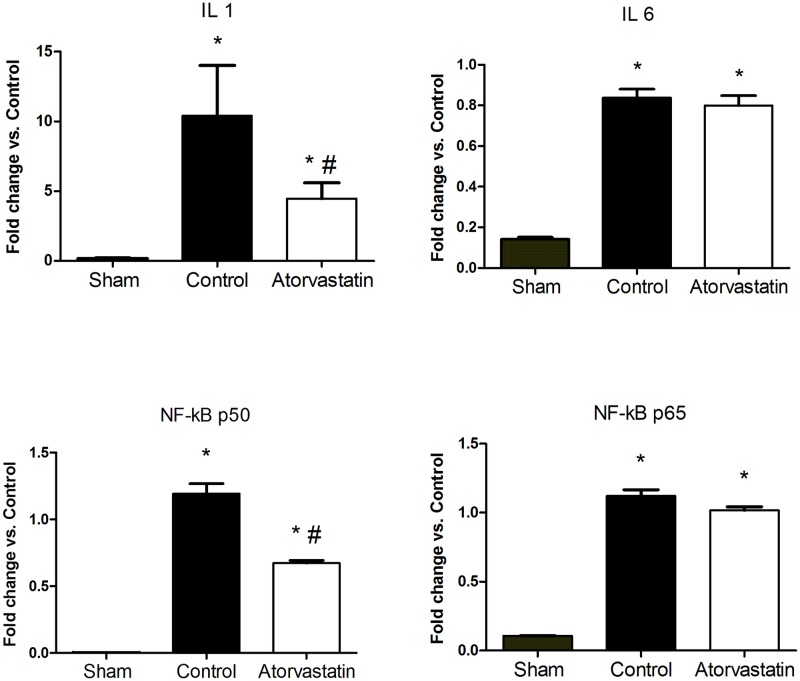
Quantitative PCR Results for mRNA Levels of Genes Related to Inflammatory Processes. Expression levels were normalized using actin as a reference gene. Data are expressed as mean ± SD.*P<0.05 versus sham group, #P<0.05 versus control group. Data representative of 8 animals in each group.

Using quantitative real-time PCR, we evaluated the anti-inflammatory properties of atorvastatin by analyzing the mRNA expression levels of genes encoding some pro-inflammatory cytokines, such as *IL-1*, *IL-6*, *NF-κB p50*, and *NF-κB p65* (Fig 6). Atorvastatin treatment significantly reduced the mRNA expression level of the *NF-κB p50* compared to the control group, but without affecting the expression of the *NF-κB p65*. Atorvastatin treatment significantly reduced mRNA levels of the *IL-1*, but did not significantly affect levels of the *IL-6*, compared to the control group.

## Discussion

In this study, we evaluated the action of atorvastatin in collagen metabolism and ventricular remodeling using a rat model of MI. Our main finding was that atorvastatin treatment for 4 weeks helped to prevent or mitigate these deleterious effects.

After tissue injury, production and deposition of collagen are increased in the ECM, leading to fibrosis, heart stiffness, as well as systolic and diastolic dysfunctions [30]. Atorvastatin treatment reduced the fibrotic area in the LVs of rats. We were able to demonstrated a reduction of fibrosis, but not collagen deposition assessed by histological qualitative analyses. The Masson trichrome permits a quantitative analysis of all types of collagen and extracellular matrix components in a sample. Whereas, the Picrusirius red is more specific for staining types I and III collagen molecules. However, the protein expression assessed by immunoblotting revealed lower content of collagen I and similar levels of collagen III.

Atorvastatin was also effective in maintaining the ventricular integrity and function, as demonstrated by the lower values of ESV and EDV. Previous studies using animal models showed that the systolic and diastolic functions of the heart can be preserved and enhanced with the use of other statins, such as pravastatin and fluvastatin [13, 31]. Seven statin types are currently available that can potentially provide similar effects and properties to atorvastatin [32]. Atorvastatin treatment before and after induction of MI can attenuate ventricular dysfunction by improving Max dP/dt, EDP, and PRSW [9]. Consistent with these findings, we observed that PRSW and ESPVR were higher after atorvastatin treatment (albeit without significant differences between the groups), indicating that atorvastatin treatment minimized contractile dysfunction and improved heart stiffness.

We evaluated the possible influence of atorvastatin on collagen metabolism after MI. MMPs are zinc-dependent endopeptidases. After activation in the myocardium, MMPs regulate ECM structure through the degradation of ECM components, particularly collagen [33], and contribute to ventricular remodeling [34, 35]. TIMPs are endogenous regulators of the catalytic action of MMPs. TIMPs regulate the processes of inflammation, dilation, and ventricular dysfunction [36]. Expression of TIMP1 after ischemic insult is fundamental to ensuring maintenance of the ventricular geometry. Increased expression levels of MMPs and TIMPs may be involved in the reduced development of fibrosis after 4 weeks of atorvastatin treatment. This effect occurs because the rate of collagen degradation is greater than the rate of synthesis, leading to reduced collagen deposition in the ECM and less cardiac fibrosis [23].

In the present study, we found that atorvastatin treatment significantly increased expression levels of MMP-1/8 and TIMP1. Atorvastatin exerted this effect on collagen synthesis via two specific markers: PCPE and SPARC. PCPE is a glycoprotein involved in enhancement of procollagen C proteinases, which are key enzymes in the synthesis of new collagen molecules [37]. SPARC is a matricellular protein that is expressed after pathological events, such as MI, fibrosis, and hypertrophy [38]. SPARC modulates the formation of new collagen fibers through its binding to procollagen molecules [39]. SPARC levels are decreased in the normal adult heart, where as expression levels are elevated after tissue injury [40]. We found that atorvastatin negatively influenced expression levels of PCPE, SPARC, and COL I, but we did not observe any effects in relation to COL III. Taken together, our findings of increased expression levels of MMP1 and TIMP1 and reduced expression levels of PCPE, SPARC, and COL I indicate that atorvastatin strongly increased the degradation and decreased the synthesis of collagen, resulting in lower fibrosis content. We believe that the potent effect of atorvastatin on collagen metabolism was also reflected in the improvement of ventricular function (i.e., lower EDV).

A consequence of ventricular remodeling after MI, cardiac hypertrophy is directly associated with heart failure [41]. Statins have anti hypertrophic effects through mediation of the peroxisome proliferator-activated receptor (PPAR) pathway [42]. PPARs inhibit cardiac hypertrophy by reducing pro-inflammatory cytokine levels [42, 43]. Prior studies demonstrated that atorvastatin prevents the reduction of PPAR levels and, thereby, limits cardiac hypertrophy [44, 45]. We evaluated the degree of hypertrophy through the myocyte cross-sectional area, which was considerably reduced in animals treated with atorvastatin. Although we did not study the PPAR pathway, we found that atorvastatin reduced the development of hypertrophy through its anti-inflammatory properties. PPARs may influence these effects.

After an initial ischemic insult, several inflammatory mediators are activated and stimulated to move to the site of tissue injury [19]. Infiltrating cells (i.e., macrophages, monocytes and neutrophils) secrete cytokines that exacerbate the inflammatory response [20], which may induce an expansion of the infarct to remote regions of the LV.

Beyond stabilizing atherosclerotic plaques, statins can improve endothelial function and reduce cytokine expression through their pleotropic effects [9, 21, 22]. We evaluated the anti-inflammatory properties of atorvastatin by studying expression levels of some pro-inflammatory cytokines at remote regions of the LV. Treatment with atorvastatin induced a reduction in the levels of IL-1, but not of IL-6, in remote infarct regions. Reduced IL-1 levels were accompanied by reduced phosphorylation of IκBα. IL-1 is a pro-inflammatory cytokine responsible for some deleterious consequences after infarction, such as exacerbation of the inflammatory response, apoptosis of cardiomyocytes, fibrosis, and cardiac hypertrophy [18, 46]. Cardiomyocyte apoptosis leads to activation of the complement system and toll-like receptors (TLRs), as well as the generation of free radicals that stimulate nuclear factor kappa B(NF-κB) in cardiomyocytes, thereby inducing expression of cytokines, chemokines, and adhesion molecules [47]. NF-κB is regulated through the phosphorylation and degradation of IκB proteins, especially IkBα, which are responsible for the cytoplasmic activation and nuclear translocation of the NF-κB heterodimer p50/RelA [48–50]. Anoxic events, reactive oxygen species, and pro- inflammatory stimuli mediated by TNF-α and IL-1 can lead to the activation of the IκB kinases [48, 51–53]. In the nucleus, NF-κB p50/RelA acts as a transcription factor stimulating the expression and release of TNF-α, IL-1, IL-6, acute phase proteins, and adhesion molecules, such as ICAM1 and YCAM1, all of which are involved in the inflammatory response [53]. Thus, the beneficial effects of atorvastatin treatment may relate to its induction of reduced IL-1 levels, with concomitantly reduced stimulation of IkBα and, consequently, inhibition of NF-κB p50 expression. Although we did not observe changes in NF-κB p65 expression after atorvastatin treatment, we did observe the inhibition of NF-κB p50, associated with improvements in ventricular remodeling after MI. Absence of NF-κB p50 in knockout mice after MI can attenuate ventricular remodeling [54]. Atorvastatin was reported to inhibit NF-κB, potentially due to reduced stimulation of cytoplasmic IκBα [55–57,58].

TLRs are pattern recognition receptors expressed by leukocytes after ischemic injury in the heart [59–61]. TLRs regulate the inflammatory response by mediating the translocation of NF-κB and the release of inflammatory cytokines, such as IL-1, IL-6, and TNFα [61, 62]. TLR2 and TLR4 are the most-studied TLRs in cardiac tissue [63]. Atorvastatin treatment significantly reduced expression levels of TLR4, but not of TLR2, in our rat model of MI. From this finding, we infer that reductions in TLR4 levels were responsible for inducing lower expression levels of IL-1 and, consequently, of pIκBα and NF-kB p50. Our results are similar to the findings of Yang *et al*.,after fluvastatin treatment [62].

TN-C is a matricellular protein and an important marker of inflammation. TN-C acts on the formation of new collagen molecules and regulates NF-kB activation [6]. Absence of TN-C attenuates cardiac dysfunction after MI, by improving diastolic function and reducing fibrosis development [38, 64]. We found that atorvastatin treatment can lead also to lower expression levels of TN-C, which may contribute to reduce the inflammatory response via NF-κB p50 and to minimize the progression of adverse ventricular remodeling. In our manuscript atorvastatin had a beneficial influence on ventricular remodeling. The ventricular remodeling process may occur in several different clinical scenarios such as chronic aortic regurgitation and chronic mitral regurgitation. Although we did not evaluate the atorvastatin effect on those clinical situations, we may extrapolate a potential beneficial effect with regarding of collagen metabolism. However, we do not have data for this assumption. Further clinical trials and further investigations are necessary.

Previous studies have investigated the use of atorvastatin at a dosage of 10mg/kg/day. Similarly to our findings, these previous studies showed improvement of ventricular function, reduction of collagen deposition due to lower synthesis, and reduced levels of fibrosis [9, 23]. Atorvastatin may exhibit similarities or differences compared to other statins. However, we did not perform experiments comparing atorvastatin with other statins, and further studies are needed for this purpose.

As limitation of this study we can mention that is an experimental model and not a clinical study. However, this MI experimental model has been utilized extensively reported in the literature and mimics the pathophysiological events in humans after coronary artery occlusion [65].

For the surgical procedures, isoflurane was used as an anesthetic agent. The isoflurane may attenuate the deleterious effects caused by ventricular remodeling as demonstrated by Roehl and collaborators [66]. However, all animals underwent to the same anesthesia protocol being exposed to isoflurane for a short time, approximately 2 minutes. Therefore, if there were any isoflurane effect on the left ventricle remodeling, this will be observed throughout all groups. All the results were reported in comparison to the SHAM and Control groups.

In conclusion, the administration of atorvastatin for 4 weeks may be a therapeutic strategy to modulate and limit the reparative processes of ventricular remodeling. We found that atorvastatin improved ventricular function and attenuated the fibrotic, hypertrophic, and inflammatory responses after MI, in addition to modulating collagen metabolism in the experimental model. Atorvastatin may be a treatment choice for reversing these conditions and for controlling the effects and damage caused by ventricular remodeling.
