# Correction: Atorvastatin Improves Ventricular Remodeling after Myocardial Infarction by Interfering with Collagen Metabolism

**DOI:** 10.1371/journal.pone.0172453

**Published:** 2017-02-14

**Authors:** Karla Reichert, Helison Rafael Pereira do Carmo, Anali Galluce Torina, Daniela Diógenes de Carvalho, Andrei Carvalho Sposito, Karlos Alexandre de Souza Vilarinho, Lindemberg da Mota Silveira-Filho, Pedro Paulo Martins de Oliveira, Orlando Petrucci

The fifth author’s name appears incorrectly in the citation. The fifth author’s name in the citation should be Sposito AC. The correct citation is: Reichert K, Pereira do Carmo HR, Galluce Torina A, Diógenes de Carvalho D, Sposito AC, de Souza Vilarinho KA, et al. (2016) Atorvastatin Improves Ventricular Remodeling after Myocardial Infarction by Interfering with Collagen Metabolism. PLoS ONE 11(11): e0166845. doi:10.1371/journal.pone.0166845
